# Targeted delivery of budesonide in acetic acid induced colitis: impact on miR-21 and E-cadherin expression

**DOI:** 10.1007/s13346-023-01363-2

**Published:** 2023-05-15

**Authors:** Shaymaa S. Seoudi, Eman A. Allam, Amal H. El-Kamel, Hagar Elkafrawy, Riham M. El-Moslemany

**Affiliations:** 1 Department of Pharmaceutics, Faculty of Pharmacy, Alexandria University, Alexandria, Egypt; 2 Department of Medical Physiology, Faculty of Medicine, Alexandria University, Alexandria, Egypt; 3 Department of Medical Biochemistry, Faculty of Medicine, Alexandria University, Alexandria, Egypt; 4 Center of Excellence for Research in Regenerative Medicine and Applications (CERRMA), Faculty of Medicine, Alexandria University, Alexandria, Egypt

**Keywords:** pH-responsive, Nanoparticles, Hyaluronic acid, Inflammatory bowel disease, Caco-2 cells, Inflammatory markers

## Abstract

Inflammatory bowel disease (IBD) is characterized by chronic inflammation along the gastrointestinal tract. For IBD effective treatment, developing an orally administered stable drug delivery system capable of targeting inflammation sites is a key challenge. Herein, we report pH responsive hyaluronic (HA) coated Eudragit S100 (ES) nanoparticles (NPs) for the targeted delivery of budesonide (BUD) (HA-BUD-ES-NPs). HA-BUD-ES-NPs showed good colloidal properties (274.8 ± 2.9 nm and − 24.6 ± 2.8 mV) with high entrapment efficiency (98.3 ± 3.41%) and pH-dependent release profile. The negative potential following incubation in simulated gastrointestinal fluids reflected the stability of HA coat. In vitro studies on Caco-2 cells showed HA-BUD-ES-NPs biocompatibility and enhanced cellular uptake and anti-inflammatory effects as shown by the significant reduction in IL-8 and TNF-α. The oral administration of HA-BUD-ES-NPs in an acetic acid induced colitis rat model significantly mitigated the symptoms of IBD, and improved BUD therapeutic efficacy compared to drug suspension. This was proved via the improvement in disease activity index and ulcer score in addition to refined histopathological findings. Also, the assessment of inflammatory markers, epithelial cadherin, and mi-R21 all reflected the higher efficiency of HA-BUD-ES-NPs compared to free drug and uncoated formulation. We thus suggest that HA-BUD-ES-NPs provide a promising drug delivery platform for the management and site specific treatment of IBD.

**Graphical Abstract:**

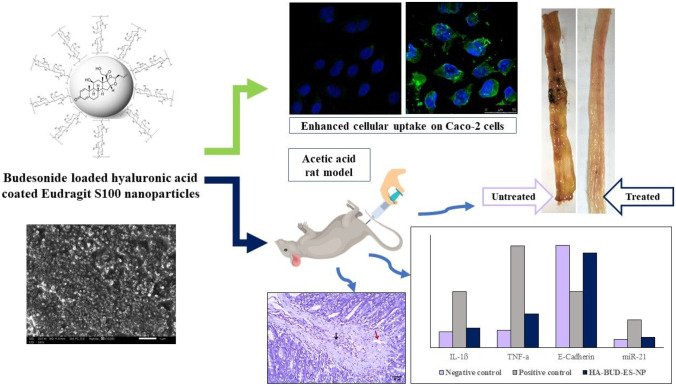

## Introduction

Inflammatory bowel disease (IBD) is one of the debilitating autoimmune diseases. It is characterized by chronic inflammation along the gastrointestinal tract (GIT) tract. IBD patients suffer from recurrent attacks of intestinal inflammation manifested by attacks of abdominal pain, diarrhea, weight loss, rectal bleeding, and anemia [1]. IBD is clinically classified into two main types: Crohn’s disease (CD) and ulcerative colitis (UC) [2]. CD or UC incidence can contribute to the risk of other diseases such as colorectal cancer [3].

IBD etiology includes many factors, such as genetics, food and pharmaceutical consumption, and GIT microbiota makeup [4]. One of the main important theories for IBD pathogenesis is the increased gut epithelium permeability [5], due to the disrupted gut epithelial barrier. This results in increased exposure to the gut luminal microbiota antigens resulting in activation of the mucosal immune system, recruiting many inflammatory cells to the GIT wall lamina propria with the release of many inflammatory mediators, along with damaging compounds such as reactive oxygen species (ROS) [6].

IBD treatment includes pharmaceuticals, antibody therapy, and even full surgical procedures. Others include corticosteroids, though their prolonged use needs close monitoring of their side effects such as osteoporosis, diabetes, and hypertension. Sometimes, immunosuppressants are prescribed for IBD patients; however, this increases the risk of drug-induced hepatotoxicity, neutropenia, and opportunistic infections [7].

The European Crohn’s and Colitis Organization (ECCO) stated that oral budesonide (BUD) is the first drug of choice in IBD treatment [8]. Budesonide is a glucocorticoid that acts locally and can be administered orally or via the rectal route using enema or suppositories [9]. After oral administration, it is subjected to 90% first-pass hepatic metabolism so only 10% of the absorbed amount reaches the systemic circulation resulting in lower systemic adverse effects compared to conventional corticosteroids [9]. Unfortunately, it is difficult to enable sufficient cellular uptake of budesonide at inflammation sites which can be due to drug degradation by gastric pH and intestinal enzymes, in addition to systemic drug absorption.

Therefore, the development of a drug delivery system (DDS) for specific targeted delivery of BUD to the inflamed area has the benefit of enhanced therapeutic efficacy while maintaining a good safety profile [10, 11]. Colloidal drug carrier systems for the targeted drug delivery to inflammatory sites for IBD therapy have recently been the focus of several studies such as micro- or nanoparticles [12–14].

Reducing nanoparticle diameter is said to increase the residence duration in the digestive tract. In addition to size, the carriers’ surface charge can have an impact on how well they target. It was established that particles with negative charges may potentially accumulate more heavily in areas where cationic proteins are overexpressed, such as on the surface and in the surrounding environment of inflammatory cells. As a result, negatively charged nanoparticles accumulate preferentially near the site of inflammation and adhere to it more firmly [15, 16]. Since passive targeting using size and charge is insufficient to enable 100% medication absorption at inflammatory locations, active targeting employing ligands for specified cells like hyaluronic acid can overcome this drawback [17, 18]. For colon-specific drug delivery, Eudragit S100 (ES100) is the most used biocompatible polymer. It has been approved for oral administration in the USA, Europe, and Japan. The polymer dissolves selectively in pH 6–7 aqueous media releasing encapsulated drug in the colon. Due to the change in intestinal pH, a large amount of encapsulated drugs is immediately released from ES100-loaded preparations [19]. Several studies have investigated the use of nanotechnology based ES100 formulations for colon specific drug delivery. To achieve this goal, ES100 was either used as a coat for various types of nanoparticles such as PLGA [20] and pectin [21] or as a polymer forming pH sensitive nanoparticles [22, 23].

Hyaluronic acid (HA) is a naturally occurring high molecular weight anionic polysaccharide. Its advantages include biocompatibility, mucoadhesion, and biodegradability [24]. It actively targets CD44 receptors, which endothelial cells overexpress at inflammatory sites in IBD as a result of enhanced cell proliferation. These benefits make hyaluronic acid an excellent choice for coating nanoparticles to selectively target inflammatory areas in IBD [25].

Considering the previously mentioned considerations, the study’s major goal was to develop and evaluate colon specific oral BUD-loaded ES100-nanoparticles coated with hyaluronic acid that target inflammation sites in IBD. Scanning electron microscopy (SEM), particle size (PS), drug loading (DL%), and encapsulation efficiency (EE%) were used to characterize the NP system. In addition, in vitro drug release characteristics were studied. In vitro studies on Caco-2 cell line were also conducted. Following optimization, in vivo targeting potential of the selected formulation was evaluated. Acetic acid-induced colitis rat model was selected. It is worth mentioning that, as far as we know, this is the first research assessing the therapeutic effectiveness of hyaluronic-coated BUD-ES100 NPs against an acute experimental rat model of IBD.

## Materials and methods

### Materials

Eudragit S100 (ES100) was from Evonik Industries AG (Essen, Germany). Budesonide (BUD), hexadecyltrimethyl ammonium bromide (CTAB), and poloxamer 188 were purchased from Sigma Aldrich (USA). Hyaluronic acid sodium salt (Mwt 1.4*10^6^ Da) was provided by Euromedex (Strasbourg, France). HPLC grade solvents were from Fisher Scientific (UK). Pepsin and pancreatin were also purchased from Sigma Aldrich (USA). All other chemicals and organic solvents were of analytical grade.

### Preparation of budesonide-loaded Eudragit S100 nanoparticles (BUD-ES-NP)

Budesonide (BUD)-loaded ES100 nanoparticles (ES-NP) were prepared as reported with some modifications [26]. ES100 and BUD in the ratio of 4:1 were dissolved in acetone and then injected into an aqueous solution of poloxamer 188 (3 mg/ml) under magnetic stirring. The mixture was left to stir overnight at 400 rpm at room temperature. Dispersion centrifugation was then done at 12,000 rpm for 25 min at 4 °C to remove remaining traces of organic solvent. Nanoparticles were reconstituted in 3 ml deionized water and sonicated till complete dispersion. Positively charged nanoparticles were prepared by adding CTAB (5 mg) to the ES100/BUD solution (BUD-ES-NP +).

### Hyaluronic acid coating of ES100 nanoparticles (HA-BUD-ES-NP)

Formerly prepared CTAB/ES100 nanoparticles were added dropwise to 1 ml aqueous solution of hyaluronic acid (0.5–1 mg/ml) and left to stir for 15 min. This was followed by bath sonication for 5 min.

### In vitro characterization of Eudragit S100 nanoparticles

#### Colloidal properties

The z-average particle size (PS) and polydispersity index (PDI) of ES-NP formulations were determined by dynamic light scattering (DLS) using Zetasizer^®^ Nano ZS series DTS 1060, Malvern Instruments S.A, Worcestershire, UK. Zeta potential (ZP) was determined at 25 °C in water (dielectric constant 79, refractive index 1.32, viscosity 0.88 cP) using a cell voltage of 150 V and 5 mA current. ES-NP dispersions were appropriately diluted with filtered deionized water before measurement to assure conveniently scattered intensity on the detector. Analyses were repeated three times (*n* = 3) [27].

#### Morphological properties

Morphological evaluation of the prepared nanoparticles was done by scanning electron microscopy (SEM). BUD-ES-NP + and HA-BUD-ES-NP were mounted on aluminum and sputter-coated with a thin layer of gold and shots were taken at × 15 K at 30 kV [28].

#### Determination of budesonide entrapment efficiency

BUD entrapment efficiency (EE) was determined by measuring free BUD concentration in the supernatant following centrifugation of the NP dispersion at 12,000 rpm for 25 min at 4 °C. BUD concentration in the supernatant was quantified by a previously reported HPLC–UV assay [29]. Agilent Technologies-1260 infinity; Santa Clara, CA, USA was used. Separation was carried out on reversed phase ZORBAX Eclipse Plus C18 column (4.6 × 150 mm, 5 μm) at room temperature. An isocratic eluent consisting of acetonitrile and phosphate buffer pH 3.2 in the ratio 65:35 v/v was used. The injection volume was 20 μl and the flow rate was adjusted to 1.5 ml/min. Peaks were detected at 243 nm using a UV detector. The retention times of BUD isomers were 12.1 and 13.2 min. BUD concentration was calculated using calibration standards based on the sum of the peak areas of the two isomers. Linearity was checked in the concentration range of 20–1000 µg/ml with a determination coefficient of 0.999. Measurements were made in triplicate. The %EE of BUD in ES-NPs was calculated from the difference between the initial drug concentration added and the free drug concentration in the supernatant using the following equation:

$$\%EE=\frac{\text{Total drug (mg)-unentrapped drug (mg)}}{\text{Total drug (mg)}}\text{x 100}$$

### In vitro drug release

The in vitro drug release profile of BUD from BUD-ES-NP + and HA-BUD-ES-NP was evaluated by the dialysis method using presoaked cellulose dialysis bags (MW cutoff 10–14 KDa). The experiment was carried out in a shaking water bath for 24 h at 100 rpm at 37 °C. Triplicate 200 μl samples of tested formulations were introduced into the dialysis bag and immersed in a 20-ml release medium. For the first 2 h, the release medium consisted of 2 mg/ml sodium chloride in pH 1.2 adjusted using hydrochloric acid. The bag was then moved to a release medium consisting of 6.8 mg/ml monobasic potassium phosphate and of pH 7.4 adjusted using 1 N sodium hydroxide for the subsequent 22 h. Sodium lauryl sulfate (SLS, 0.5% w/v) was added to both dissolution media to maintain sink conditions [30]. Samples (each of 0.5 ml) were withdrawn at predetermined time intervals and replaced with the same amount of fresh-release medium. BUD concentration was determined by the mentioned HPLC–UV method.

### Stability assessment

#### Storage stability

Colloidal stability of BUD-ES-NP + and HA-BUD-ES-NP at 4 °C was assessed at 2 weeks intervals over 2 months. Triplicate samples were stored in the refrigerator and tested by measuring particle size and zeta potential.

#### Stability in simulated gastrointestinal fluids

The gastrointestinal stability of nanoparticles was assessed by measuring particle size and zeta potential following incubation in simulated gastric (SGF) and intestinal fluids (SIF) at 37 °C. Formulations were diluted to a final concentration, 10% v/v in simulated fluids and incubated at 37 °C. Samples were measured at times 0, 1, and 2 h for SGF. For SIF, a 6-h sample was also measured. SGF and SIF were prepared according to the United States Pharmacopeia 33-28NF (2010). SGF consisted of 0.32% w/v pepsin, 2 mg/ml sodium chloride, and the pH was adjusted to 1.2 using hydrochloric acid, SIF consisted of 1% w/v pancreatin, 6.8 mg/ml monobasic potassium phosphate, and the pH was adjusted to 7.4 using 1 N sodium hydroxide. Samples in SGF were neutralized to pH 7 before the measurement of zeta potential to eliminate the effect of free protons in the medium.

### In vitro studies on Caco-2 cell line

#### Cell culture

Caco-2 cells, a human epithelial colorectal cancer cell line, were purchased from ATCC (HTB-37™). The study was conducted at the Center of Excellence for Research in Regenerative Medicine and Applications (CERRMA) at Alexandria Faculty of Medicine. Cells were grown as monolayer cultures in DMEM/high‐glucose (Dulbecco’s modified Eagle medium; Lonza; containing 0.2 mmol/ml L‐glutamine) supplemented with 100 IU/ml penicillin, 100 μg/ml streptomycin Lonza, and 10% fetal bovine serum (FBS; Lonza). Cells were maintained in a humidified 37 °C, 5% CO_2_ incubator. Cells were monitored daily for their growth and morphology using the phase‐contrast inverted microscope (CKX41SF; Olympus). Media was changed every 2–3 days, and cells were passaged on reaching 80–90% confluence by suspension with 0.25% w/v trypsin‐EDTA (Lonza), then plated in T75‐cm^2^ flasks for maintenance or in 6- or 96‐well plates according to the experiment conducted.

#### Cell viability study

Cells were seeded in 96-well flat-bottom plates at a density of 7 × 10^3^ cells per well and incubated for 24 h. The medium was replaced by fresh medium containing different concentrations (0.1–3.2 μg/ml) of the formulations and incubated for 48 h. The concentration of dimethyl sulfoxide (DMSO) in the medium was kept at < 0.1%. The methylthiazolyldiphenyl-tetrazolium bromide (MTT) was used to evaluate the in vitro cytotoxic effect of free BUD, BUD-loaded and unloaded ES-NPs and HA-ES-NPs nanoparticles on Caco-2 cells. After 48 h incubation at 37 °C and 5% CO_2_, the culture media were aspirated, replaced by 100 μl of new media with (0.5 mg/ml) of MTT solution, and incubated for 4 h. After incubation, the media was removed and 100 μl DMSO/well was added and gently rocked in the dark by an ELISA shaker for 20 min. The absorbance was measured at 570 nm by ELISA well-plate reader (Tecan, Infinite F50). The percentages of cell viability were calculated as the ratio of treated to untreated cell absorbances.

#### Determination of in vitro cellular uptake

Cellular uptake of coumarin-6 (C6)-loaded ES-NP + and HA-ES-NP compared to free C6 solution was investigated by confocal laser scanning microscopy [27]. C6-labeled formulations were prepared by the procedure mentioned in the “Preparation of budesonide-loaded Eudragit S100 nanoparticles (BUD-ES-NP)” and “Hyaluronic acid coating of ES100 nanoparticles (HA-BUD-ES-NP)” sections replacing BUD with C6 at a concentration of 200 μg/ml. C6-loaded nanoparticles and free solution were added to Caco-2 cells at a final concentration of 5 μg/ml and then incubated at 37 °C for 4 h. Cells were washed with PBS, fixed with 4% paraformaldehyde for 10 min, and then washed twice with 2 ml PBS. Cells were permeabilized with triton X (0.2%) for 10 min, then washed twice with 2 ml PBS and stained with 4′,6-diamidino-2-phenylindole (DAPI). Confocal laser scanning microscopy (LEICA, DMi8, Mannheim/Wetzlar, Germany) equipped with an argon laser was then used for imaging. C6 and DAPI-stained cell nuclei were observed through the blue and green channels at 405 and 485 nm excitation, respectively. Cellular uptake was determined through the assessment of the fluorescence intensity of 15 cells from 3 different images by ImageJ software (Version 1.52).

#### In vitro assessment of the anti-inflammatory effect

Induction of inflammation was done by exposing cells to the pro-inflammatory lipopolysaccharide (LPS, Sigma Aldrich, USA) at 1 μg/ml for 48 h at 37 °C [31]. To investigate the presence of inflammation in the cell model, extracellular media were collected to analyze the secretion of IL-8 and TNF-α using enzyme-linked immunosorbent assay (ELISA) kits (Elabscience, USA, Cat. No: E-EL-H6008) and (Cusabio, USA, Cat. No.CSB-E04740h) according to manufacturer’s instructions. The anti-inflammatory efficacy was assessed for BUD-ES-NP + and HA-BUD-ES-NP compared to free drug solution and cell controls (untreated control and LPS treated control). Measurements were performed in triplicate.

### In vivo studies

#### Animals

Male Wistar rats (25 rats, weight 220 ± 20 g) were provided by the animal facility of the Faculty of Medicine, Alexandria University. Rats were housed at room temperature with ad libitum access to food and water and were maintained at a regular 12/12-h light/dark cycle. Animals were acclimatized for 5 days before the experiment. The protocol was approved by the Institutional Animal Care and Use Committee, Faculty of Pharmacy, Alexandria University (Approval number: AU-06–2022/130).

#### Induction of colitis and experimental design

Prior to colitis induction, 20 rats were fasted for 24 h with free access to water. Colitis was then induced by rectal administration of 2 ml of 4% acetic acid solution using a flexible plastic cannula inserted 4 cm into the colon under light anesthesia [23]. Ensuing acetic acid administration, rats were held upside down in a horizontal position for 1 min to prevent leakage. To allow the development of colitis, rats were left for 24 h with free access to food and water before treatment. Rats were then randomly divided into 4 groups of 5 rats each as follows: untreated control (positive control), BUD suspension, BUD-ES-NP + , and HA-BUD-ES-NP. Treated groups received volumes equivalent to 500 µg/ml BUD on an empty stomach once daily for 5 consecutive days [23]. The fifth group received only a regular diet and water and was left without disease induction nor treatment and served as a negative control. Rats were sacrificed 24 h after administration of the last dose. The entire colon was immediately excised and washed with saline, and the colon length and weight were recorded. Then, the colon was opened longitudinally for digital imaging and subsequently divided into two parts, one was kept in 10% formalin for histopathological assessment while the other was homogenized in phosphate buffer saline (PBS) containing protease inhibitor, centrifuged at 3000 rpm at 4 °C for 15 min and the supernatant kept at − 80 °C for further biochemical analyses.

#### Disease activity index assessment (DAI)

Rats were observed for weight loss, stool consistency, and bleeding. Scores were determined as shown in Table 1 following a previously described protocol [32]. The sum of scores of the 3 parameters (0–4) represents the DAI which ranges from 0 (healthy) to 12 (maximal severity of colitis).

**Table 1:** Disease activity index (DAI) assessment

Score	Percentage weight loss	Consistency of stool	Bleeding
0	< 1%	Normal	Normal
1	1–5%	Normal	Occult blood ( +)
2	5–10%	Loose stool	Occult blood (+ +)
3	10–15%	Loose stool	Occult blood (+ + +)
4	> 15%	Diarrhea	Gross bleeding

#### Assessment of macroscopic ulcer score

Excised colons were visually examined, and the gross macroscopic injury was assessed using the modified ulcer scoring system previously reported by El-Tanbouly et al. [32]. Colon mucosal damage index (CMDI) was assessed on a scale of 0–10 as shown in Table 2.

**Table 2:** Colon mucosal damage index (CMDI) for assessment of macroscopic injury

Score	Description
0	Normal appearance
1	Focal hyperemia without ulceration
2	Ulcer but with no significant inflammation
3	Ulcer with one site of inflammation
4	Two or more ulcers with inflammation at two or more sites
5	Major sites of ulceration and inflammation extending more than 1 cm along colon length
6–10	Major sites of ulceration and inflammation extend more than 2 cm along the colon. The score was increased by 1 for each additional cm of involvement

#### Histopathological examination of colon tissues

Colon specimens were collected, fixed in 10% formalin saline then embedded in paraffin wax. Tissue Sects. (5 μm) were cut and stained with hematoxylin and eosin (H&E). Histopathologic evaluation was done using a light microscope (Olympus America Inc., USA) equipped with a Spot digital camera with a numerical aperture of a high resolution (16-bit digital camera (1280 × 1024) pixels). Scoring was conducted according to Wallace and Keenan [33]. Briefly, *score 0* means intact tissue with no apparent colonic damage; *score 1* means that damage is limited to surface epithelium; *score 2* means the presence of focal ulceration limited to the mucosa; *score 3* denotes the presence of focal, transmural inflammation and ulceration; *score 4* denotes the presence of extensive transmural ulceration and inflammation bordered by moderate inflammation of the mucosa, and *score 5* denotes the presence of extensive transmural ulceration and inflammation involving the entire section.

#### Assessment of inflammatory markers

The levels of tumor necrosis factor-alpha (TNF-α) (Cat#MBS175904, MyBiosource, USA) and interleukin 1ß (IL 1ß) (Cat#MBS702717, MyBiosource, USA) in colonic tissue homogenates were determined using ELISA method according to manufacturer’s instructions.

#### Assessment of epithelial cadherin

Rat epithelial cadherin (E-cad) was measured in colonic tissue homogenate using ELISA kit (cat# CSB-E07308r, CUSABIO) according to the manufacturer’s instructions.

#### Assessment of miR-21 using real-time quantitative PCR (RT-qPCR)

Total cellular RNA was extracted from frozen tissues using the TRIzol Reagent (Invitrogen, Carlsbad, CA) according to the manufacturer’s instructions. Total RNA was then reverse transcribed using the first-strand cDNA synthesis kit (ThermoScientific, USA). The resulting cDNA was amplified using Quant studio-1 real PCR system (Applied Biosystem, Thermofisher scientific, USA). Forward and reverse primers sequence for PCR amplification of miR-21 are shown in Table 3. The RT-PCR reactions were run in triplicates with signal collection at the end of each cycle. Relative miRNA transcript levels were normalized against an internal housekeeping gene (U6 snRNA) and sample differences were determined using the comparative threshold cycle (ΔΔCt) method [31].

**Table 3:** Primers used for quantitative real-time PCR (RT-qPCR)

Gene	Forward	Reverse
miR-21	GTACCACCTTGTCGGGTAGC	Universal primer
*U6 snRNA*	CTCGCTTCGGCAGCACA	Universal primer

### Statistical analysis

The results are presented as mean ± SD of at least three independent tests. All statistical analysis was performed using (GraphPad Prism version 7, CA, USA). Statistical significance was determined using one-way analysis of variance (ANOVA) followed by Tukey’s test for post hoc pair-wise comparisons. Differences were considered significant when *p* ≤ 0.05.

## Results and discussion

### Characterization of ES100 nanoparticles

**Fig. 1:**
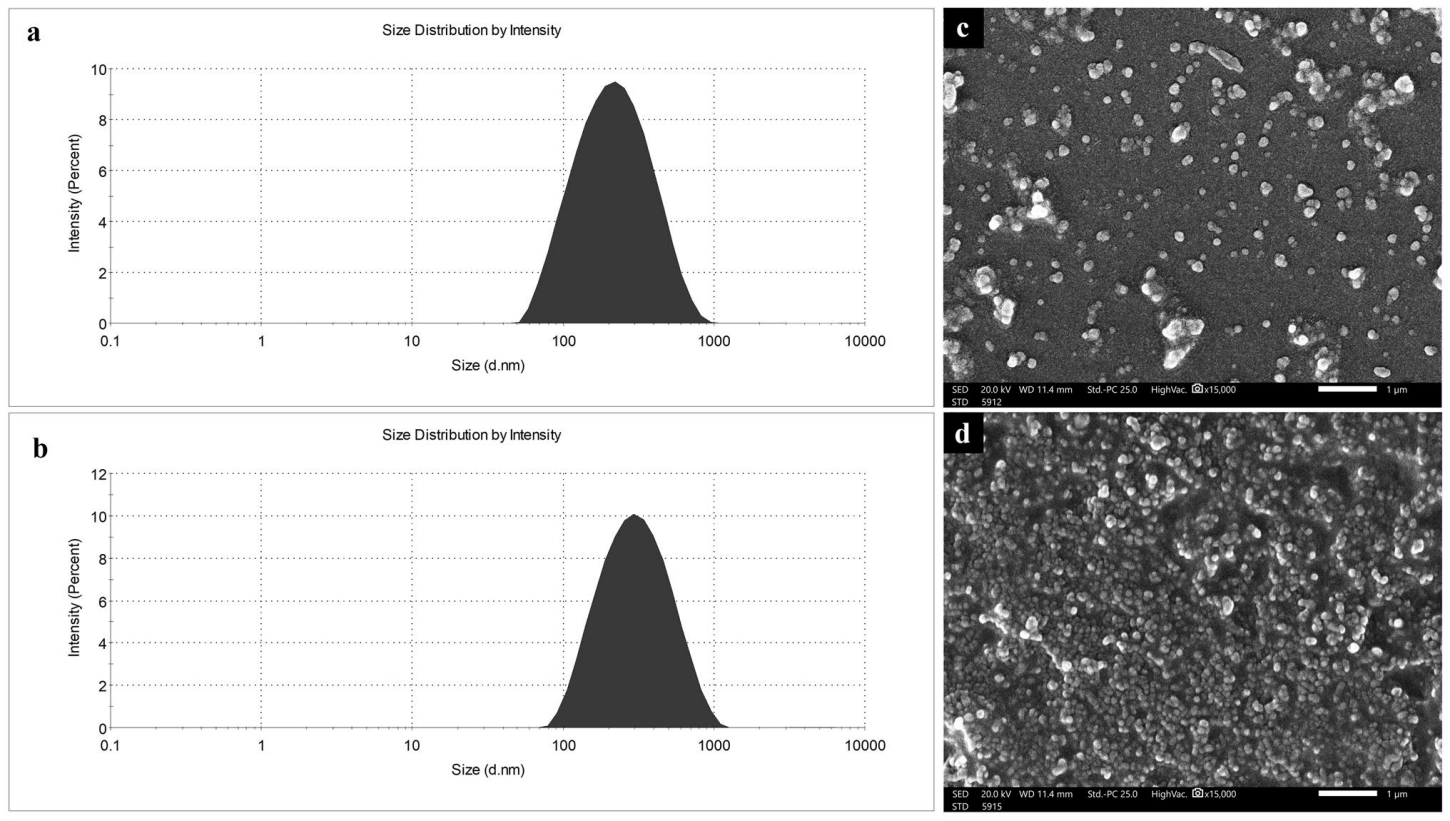
Particle size distribution by intensity curve of **a** BUD-ES-NP + and **b** HA-BUD-ES-NP and SEM images of **c** BUD-ES-NP + and **d** HA-BUD-ES-NP. Magnification × 15 K, scale bar represents 1 µm

The colloidal properties of different ES100 nanoparticles are shown in Table 4. BUD-ES-NPs were prepared with a mean size of 144.7 ± 3.2 nm. NPs showed a negative ZP (− 22.6 mV) ascribed to the characteristic dissolution and ionization of ES100 at ⁓pH 7.0 [34]. The high ZP provides an electric repulsion thus preventing particle aggregation. Positively charged NPs were prepared by adding CTAB to the formulation. A significant increase in PS (*p* ≤ 0.05) was observed with a shift to a positive ZP of 33.3 mV. The positive charge induced allowed for HA coating. This was confirmed by the increase in PS (239.5 ± 9.2 and 274.8 ± 2.9 nm for BUD-ES-NP + and HA-BUD-ES-NP, respectively) (Fig. 1a and b) and the negative charge observed following coating (− 24.6 ± 2.8 mV). For all formulations, the PDI was inferior to 0.3 which demonstrated formulation monodispersity.

**Table 4:** Physicochemical properties of ES100 nanoparticles (*n* = 3)

Formulation	Particle size (PS), nm	PDI	Zeta potential (ZP), mV	%EE
BUD-ES-NP	144.7 ± 3.2	0.213	− 22.6 ± 1.3	99.3 ± 4.21
BUD-ES-NP +	239.5 ± 9.2	0.232	33.3 ± 1.7	98.1 ± 1.65
HA-BUD-ES-NP	274.8 ± 2.9	0.278	− 24.6 ± 2.8	98.3 ± 3.41

Efficient encapsulation of BUD was attained with %EE exceeding 98% (Table 1) and an average drug payload of ⁓17.6%. The high %EE is attributed to the lipophilic nature of BUD (Log *p* value 1.9) and hence low affinity to water [35].

Morphological examination of BUD-ES-NP + and HA-BUD-ES-NP using SEM (Fig. 1c and d, respectively) showed smooth spherical nanoparticles. The size was uniform with no aggregates. HA-BUD-ES-NPs have slightly higher PS compared to the uncoated formulation. These results are comparable to those determined by DLS.

### In vitro drug release

**Fig. 2:**
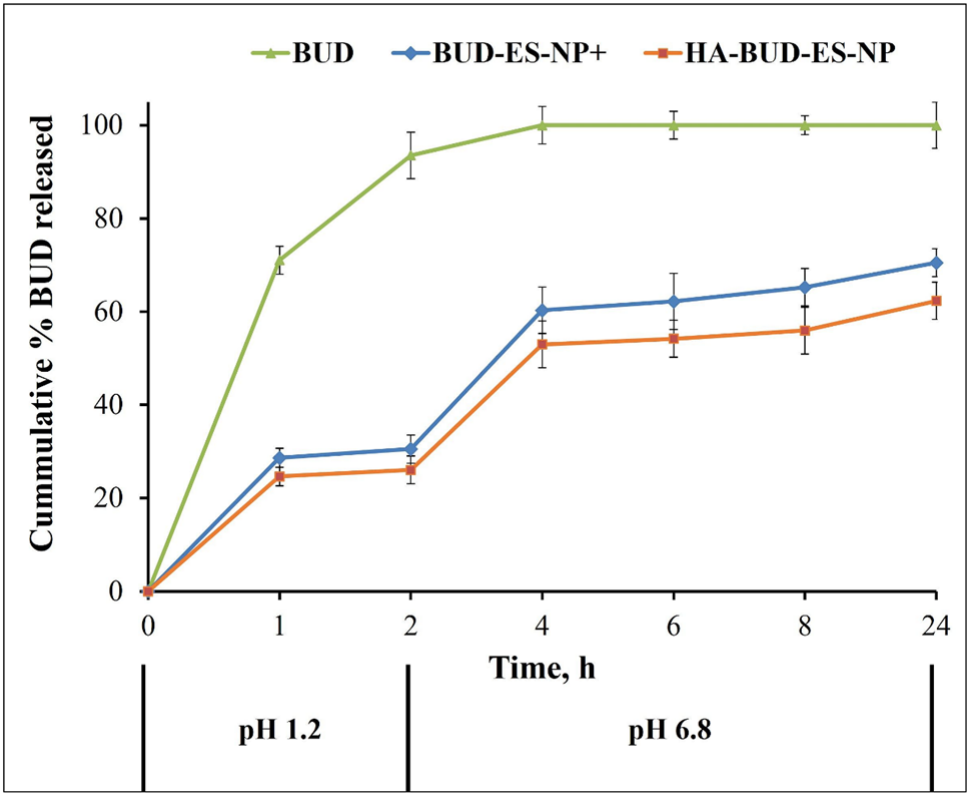
Release profile of budesonide from BUD-ES-NP + and HA-BUD-ES-NP formulations in pH 1.2 and 6.8 at 37 °C over 24 h (*n* = 3)

The pH-dependent release profile of BUD from ES100 nanoparticles under sink conditions was studied and compared to BUD suspension (Fig. 2). At pH 1.2, BUD showed a high rate of drug release; 70% during the first hour and exceeded 90% at 2-h intervals. On the other hand, BUD release from BUD-ES-NP + was highly sustained with a release of ⁓29% at 1 h probably corresponding to the surface-entrapped drug and ⁓30% after 2 h. At pH 6.8, a spurt in drug release was observed (⁓60% at 4 h). This reflects the pH-responsive dissolution of the polymer allowing for drug release at the site of inflammation. HA coating of ES-NPs resulted in a statistically insignificant (*p* > 0.5) reduction in BUD release rate compared with BUD-ES-NP + .

### Storage stability of ES100 nanoparticles

**Fig. 3:**
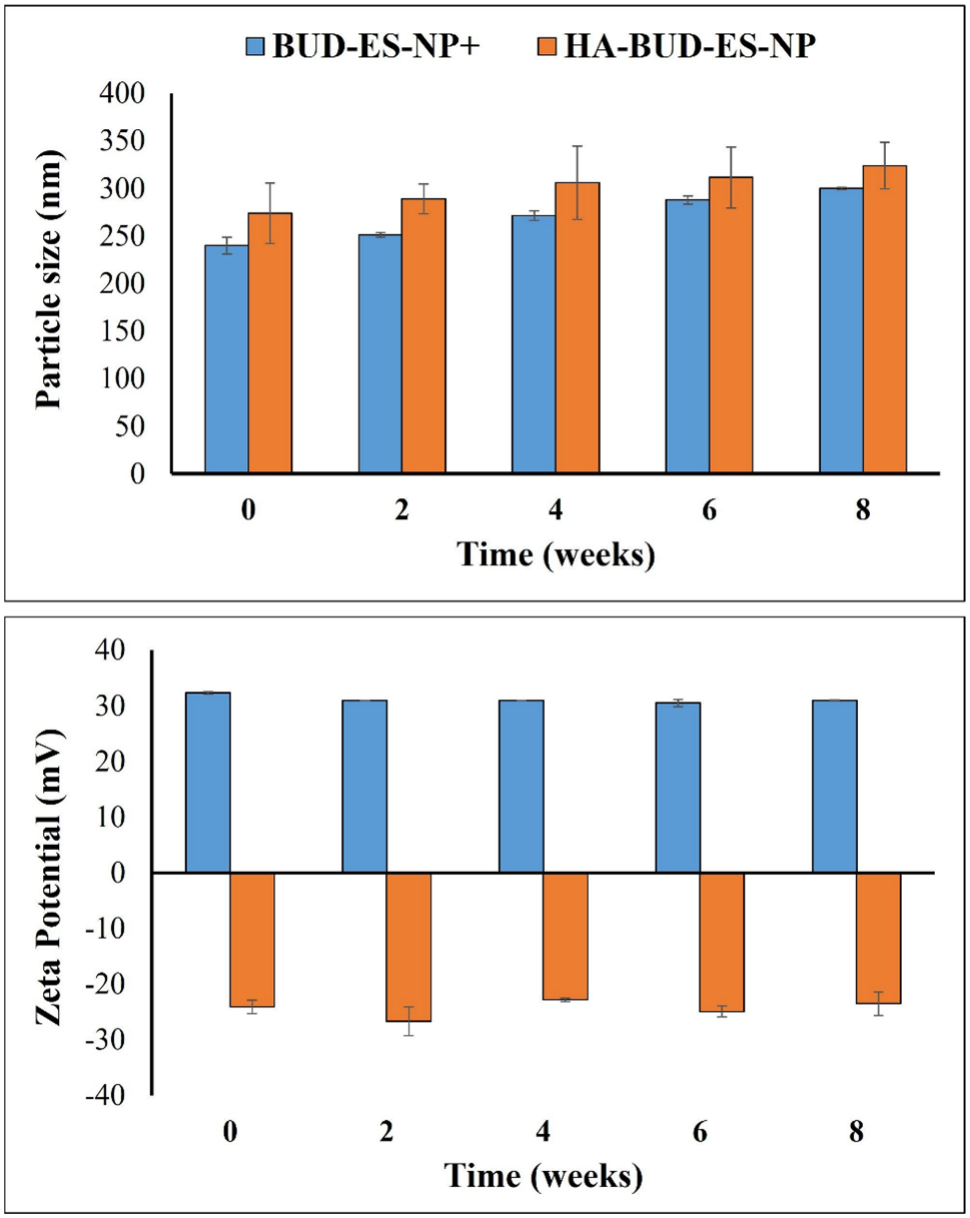
Change in particle size (nm) and zeta potential (mV) of BUD-ES-NP + and HA-BUD-ES-NP upon storage at 4 °C over 8 weeks (*n* = 3)

BUD-ES-NP + and HA-BUD-ES-NP formulations were stored at 4 °C. At 2-week intervals, PS and ZP were measured and compared to zero time (Fig. 3). Both formulations showed a slight progressive increase in size over time. In the case of BUD-ES-NP + , the increase was significant (*p* ≤ 0.05) reaching a maximum of 25% after 8 weeks. On the other hand, the HA-coated formulation increase in size was statistically insignificant (*p* > 0.05) with a maximum increase of 18%. Regarding PDI, both BUD-ES-NP + and HA-BUD-ES-NP showed an increase in PDI which remained below 0.4 indicating that the formulations relatively retained their mono-dispersity upon storage. Whereas storage affected PS, no change in ZP was observed for both formulations. Stable ZP, especially for the HA-coated formulation, reflects the stability of the coat over time which is important for targeting inflammation.

### Stability in simulated gastrointestinal fluids

In view of the planned oral administration of ES100 nanoparticle formulations, in vitro stability in simulated gastrointestinal fluids was performed (Table 5). In SGF, both formulations showed a significant increase in size (*p* ≤ 0.05). The increase was more pronounced for the HA-coated formulation (21% and 70% increase in PS for BUD-ES-NP + and HA-BUD-ES-NP after 2 h incubation, respectively). Also, a change in ZP was observed following incubation in SGF (28% and 40% decrease in ZP for BUD-ES-NP + and HA-BUD-ES-NP after 2 h incubation, respectively). A reverse pattern was observed in SIF where the change in PS and ZP was higher for BUD-ES-NP + than HA-BUD-ES-NP (109% and 37% increase in size with 56% and 49% decrease in ZP for BUD-ES-NP + and HA-BUD-ES-NP, respectively). The change in colloidal properties of ES-NPs could be explained by the formation of protein corona around the particles which is accompanied by alteration in size and surface charge [36]. This interaction is mainly driven by hydrogen bonds and van der Waals forces as previously described by Wang et al. [36]. The composition of protein corona is influenced by particle size, shape, and surface properties such as zeta potential, hydrophobicity, and functional groups [37] thus explaining the difference in behavior observed between BUD-ES-NP + and HA-BUD-ES-NP. One of the main aims of the experiment was to investigate the stability of the HA coat in gastrointestinal fluids. Retention of negative surface charge following incubation in simulated gastrointestinal fluids reflects the stability of the HA coat on the surface of positively charged BUD-ES-NP + .

**Table 5:** Stability of ES100 nanoparticles in simulated gastric (SGF) and simulated intestinal (SIF) fluids in terms of change in particle size (PS) and zeta potential (ZP) (*n* = 3)

Time (h)	BUD-ES-NP +				HA-BUD-ES-NP			
	**SGF**		**SIF**		**SGF**		**SIF**	
	PS, nm	ZP, mV	PS, nm	ZP, mV	PS, nm	ZP, mV	PS, nm	ZP, mV
**0**	240 ± 3.4	34 ± 1.6	240 ± 3.4	34 ± 1.6	277 ± 3.1	− 25 ± 3.7	277 ± 3.1	− 25 ± 3.7
**1**	263 ± 11.5	29 ± 0.7	328 ± 7.7	20 ± 0.7	365 ± 7.2	− 20 ± 2.3	269 ± 273	− 21 ± 7.1
**2**	292 ± 2.5	25 ± 3.3	448 ± 3.8	18 ± 1.1	470 ± 14.3	− 15 ± 0.2	317 ± 22.2	− 18 ± 6.2
**3**			503 ± 5.7	17 ± 0.8			378 ± 15.6	− 15 ± 2.8
**6**			ND	15 ± 1.3			ND	− 13 ± 2.3

### In vitro cell culture studies

#### Caco-2 cell viability assay

**Fig. 4:**
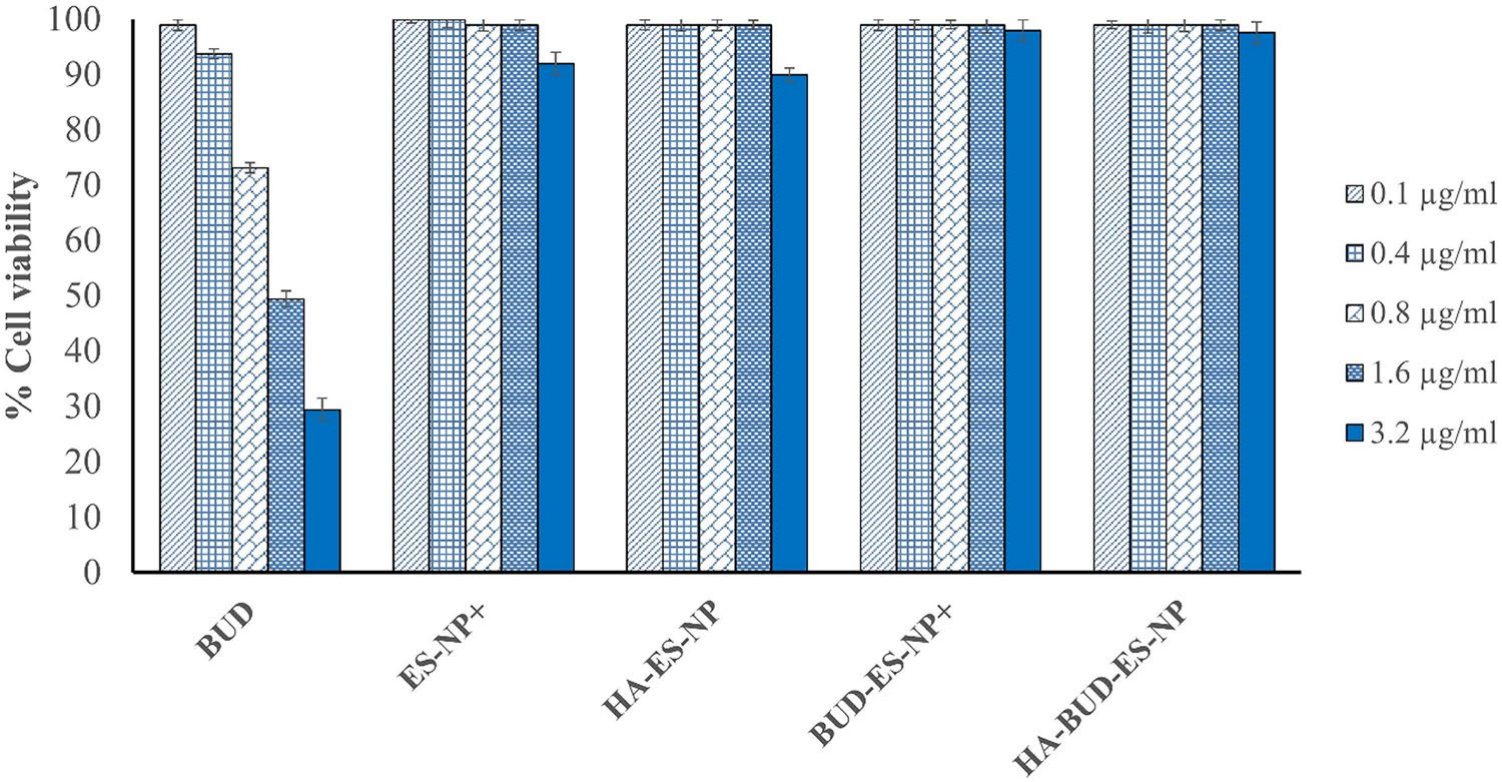
Percent Caco-2 cell viability after exposure to BUD-ES-NP + and HA-BUD-ES-NP compared to blank formulations and BUD solution. Data expressed as mean ± SD (*n* = 3)

Caco-2 cells were exposed to blank formulations and to ES100 formulations standardized at increasing BUD concentrations. Cell viability was calculated relative to control (Fig. 4). The drug solution showed dose-dependent toxicity on Caco-2 cells. As the concentration of the drug solution increased from 0.1 to 3.2 µg/ml, the cell viability decreased from 99 to 30%. On the other hand, in the drug concentration range tested (0.1–3.2 µg/ml), BUD-ES-NP + and HA-BUD-ES-NP showed cell viability exceeding 90% as obtained for blank formulation. The results reflect that loading of BUD into ES-NPs reduced drug cellular toxicity and enhanced safety on intestinal cells.

#### Cellular uptake

**Fig. 5:**
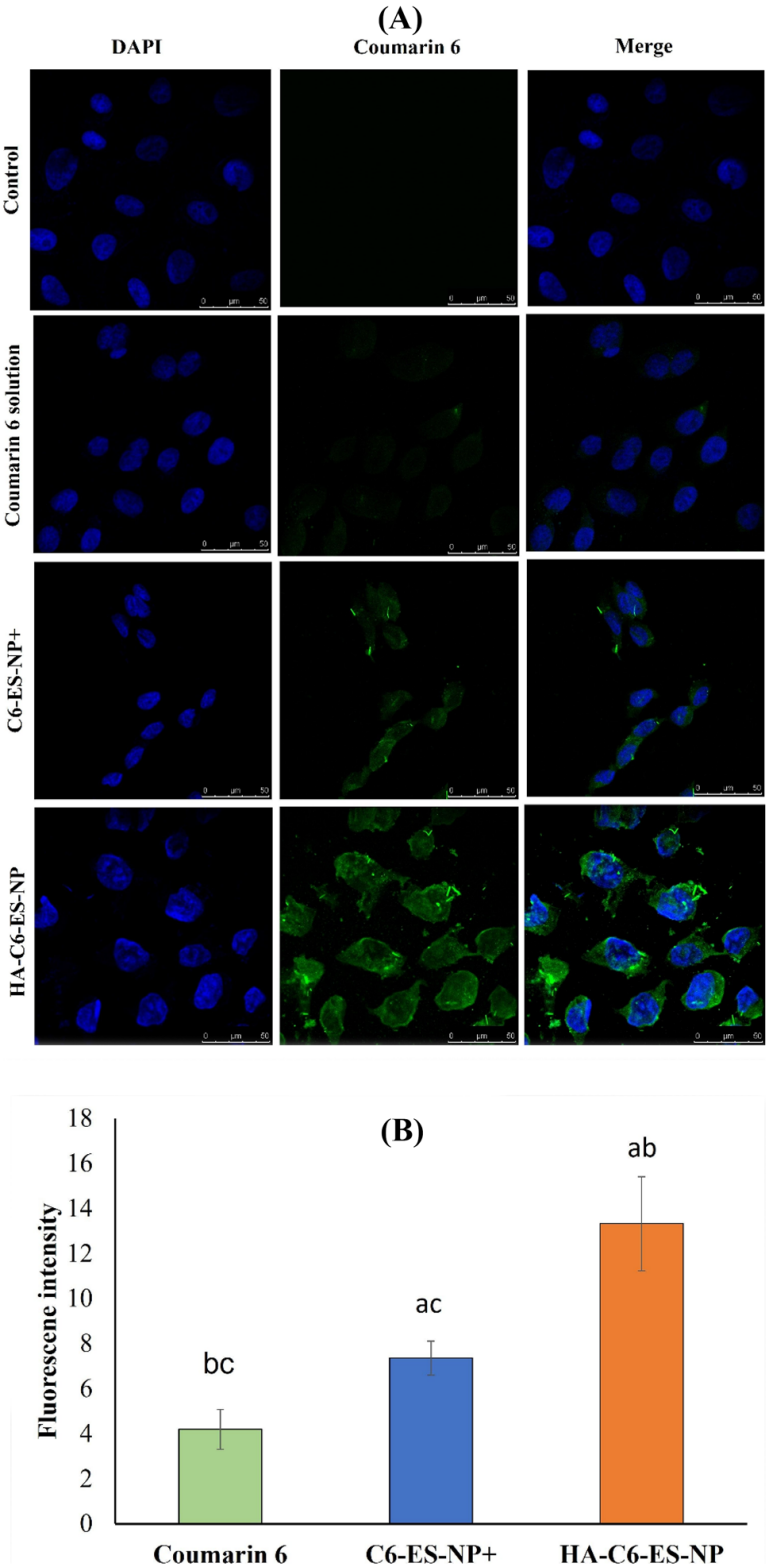
Cellular uptake of C6-ES-NP + , HA-C6-ES-NP, and coumarin-6 solution in Caco-2 cells: **A** confocal laser scan microscope images. Blue and green fluorescence signals represent the cell nuclei (DAPI) and coumarin-6, respectively, and **B** fluorescence intensity is calculated using ImageJ software. Data expressed as mean ± SD. ^a^*p* ≤ 0.05 vs coumarin-6, ^b^*p* ≤ 0.05 vs C6-ES-NP + , and ^c^*p* ≤ 0.05 vs HA-C6-ES-NP

Confocal laser microscopy scan was used to study cellular uptake of C6-loaded formulations in Caco-2 cells compared to free dye solution (Fig. 5A). Green fluorescence corresponds to C6 whereas the cell nuclei marker, DAPI, is observed as blue fluorescence signals. A quantitative assay of cellular uptake was done by calculating cellular fluorescence intensity via ImageJ software (Fig. 5B). C6 solution showed sparse fluorescence signals. Reduced Caco-2 uptake of C6-free solution has been previously reported and attributed to the inability of raw C6 to be directly internalized by the cells [38]. Loading of C6 into ES-NPs resulted in significantly higher fluorescence intensity (*p* ≤ 0.05). Moreover, the HA coating of the nanoparticles showed a further increase in fluorescence intensity in the cytoplasmic and nuclear areas. The results support the assumption that nanoparticle physicochemical properties and surface nature in contact with cells or cellular components affect cellular uptake [38, 39]. This is ascribed to the difference in cell penetration mechanisms [40]. Hence, surface modification of nanoparticles could successfully provide enhanced cellular interaction [41]. C6-ES-NP + are mostly internalized by endocytosis and passive targeting [40]. Also, the positive charge on their surface could benefit the interaction with the negatively charged cell membrane [42]. HA coating of the nanoparticles significantly improved cellular uptake which is mostly due to HA interaction with its major cell surface receptor CD44, consequently, resulting in nanoparticle internalization via receptor-mediated endocytosis. The specific affinity of HA to CD44 makes it an ideal targeting moiety for increasing cellular uptake and concentration of drugs at the surface of cancerous or inflamed colonic cells overexpressing this receptor [40, 43].

#### Inflammatory markers expression

**Fig. 6:**
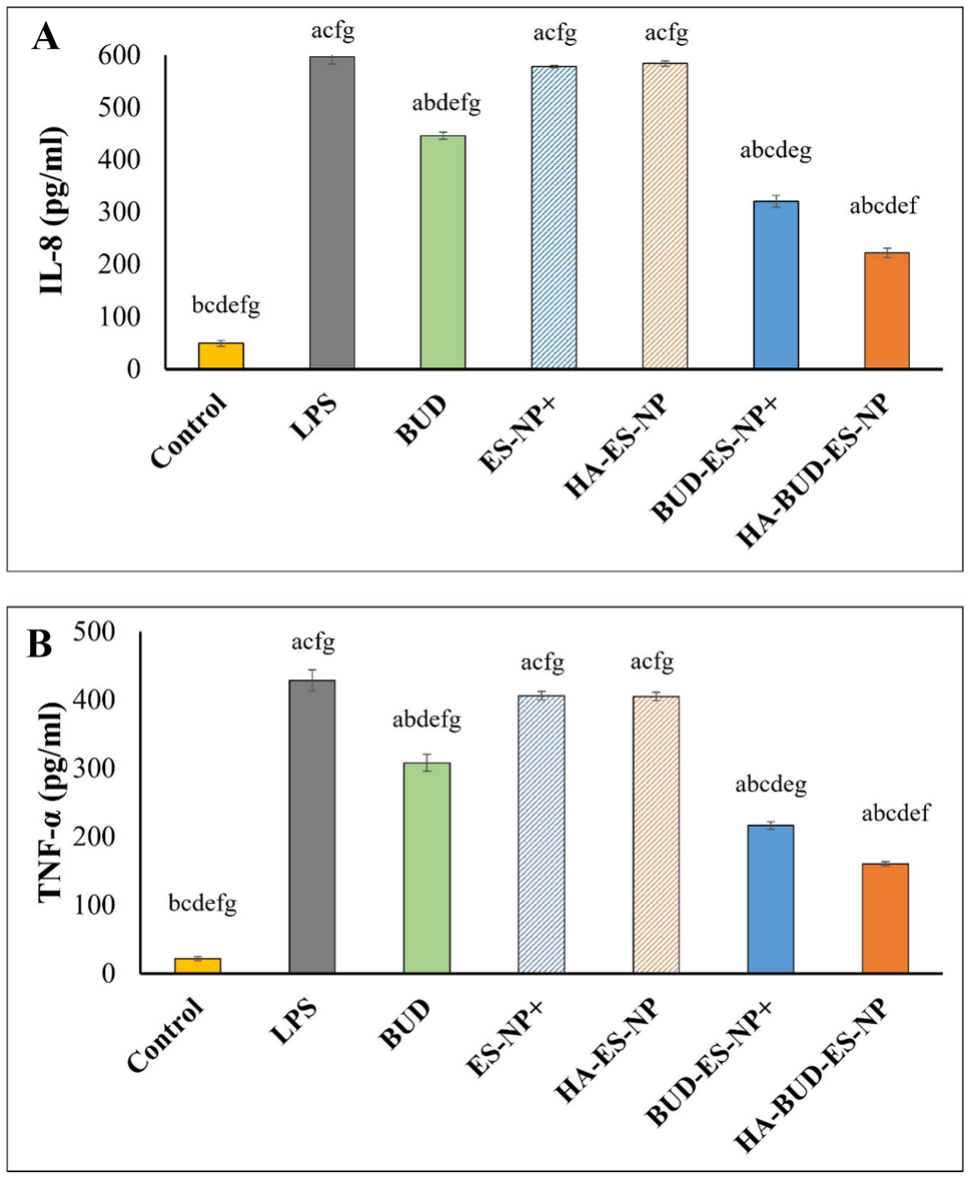
Evaluation of inflammatory markers; **A** interleukin-1ß (IL-1ß) and **B** tumor necrosis factor-alpha (TNF-α) following inflammation induction in Caco-2 cells using LPS and treatment with BUD, BUD-ES-NP + , and HA-BUD-ES-NP compared to suitable controls. Values were expressed as mean ± SD (*n* = 5). ^a^*p* ≤ 0.05 vs control, ^b^*p* ≤ 0.05 vs LPS, ^c^*p* ≤ 0.05 vs BUD, ^d^*p* ≤ 0.05 vs ES-NP + , ^e^*p* ≤ 0.05 vs HA-ES-NP, ^f^*p* ≤ 0.05 vs BUD-ES-NP + , and ^g^*p* ≤ 0.05 vs HA-BUD-ES-NP

Lipopolysaccharide (LPS) is considered one of the most potent inducers of gut inflammation [44]. It was shown that LPS can initiate a cascade of signal transduction through Toll-like receptor 4 extracellular domain binding, thus enhancing pro-inflammatory cytokine production [45] and hence is commonly used to induce cellular inflammation. Following induction of inflammation in Caco-2 cells, IL-8 and TNF-α production in the culture medium were determined using ELISA kits. As shown in Fig. 6, LPS resulted in a marked increase in IL-8 and TNF-α levels (12 and 20-fold increase, respectively, compared to LPS untreated cells). BUD treatment resulted in a significant decrease in cytokines levels (25% and 28% decrease in IL-8 and TNF-α, respectively, *p* ≤ 0.05) as it exerts a direct anti-inflammatory effect on intestinal epithelial cells [46]. Loading of BUD into ES-NPs + resulted in a further decrease in IL-8 and TNF-α levels (46% and 50%, respectively, *p* ≤ 0.05). Maximum reversal of LPS inflammatory effect was observed following treatment with HA-BUD-ES-NP (63% decrease in both IL-8 and TNF-α compared to LPS-treated cells). This could be explained by the enhanced cellular uptake achieved by the HA-coated NPs. Similarly, HA-functionalized polymeric nanoparticles [47] and BUD-loaded self-assembled HA-NPs [48] demonstrated higher anti-inflammatory effect as was shown by the reduced IL-8 and TNF-α secretion in inflamed cell models.

### In vivo efficacy

Acetic acid-induced colitis shares several clinical features of human IBD. It mimics human pathophysiology in cytokine profile and histopathological attributes and is characterized by infiltration of neutrophils and subsequent colon tissue damage via reactive species formation [49, 50]. Also, its simplicity, reproducibility, and the rapid appearance of inflammation and clinical parameters make it a popular experimental model for IBD [51].

#### Effect on colon length, DAI, and macroscopic ulcer score

**Fig. 7:**
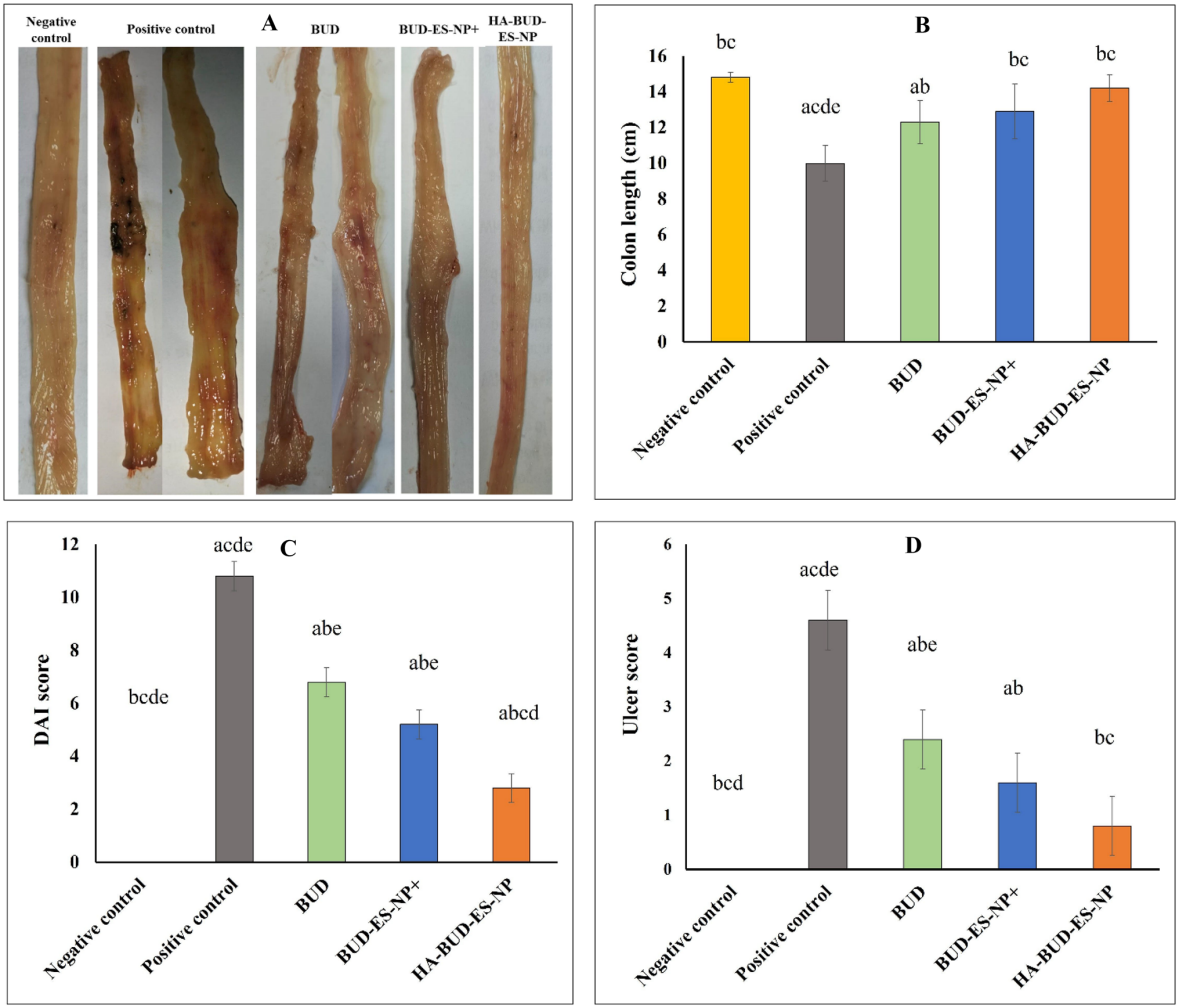
**A** Macroscopic evaluation of colitis, **B** colon length, cm, **C** disease activity index (DAI), and **D** ulcer score following treatment of acetic acid-induced colitis with BUD, BUD-ES-NP + , and HA-BUD-ES-NP compared to suitable controls. Values were expressed as mean ± SD (*n* = 5). ^a^*p* ≤ 0.05 vs negative control, ^b^*p* ≤ 0.05 vs positive control, ^c^*p* ≤ 0.05 vs BUD, ^d^*p* ≤ 0.05 vs BUD-ES-NP + , and ^e^*p* ≤ 0.05 vs HA-BUD-ES-NP

IBD induction by acetic acid led to a significant decrease in colon length (10 ± 1.5 cm) associated with a significant increase in DAI (5.4 ± 0.5) and macroscopic ulcer score (4.6 ± 0.5) in the positive control group versus the negative control (14.8 ± 0.2 cm, 0, 0, respectively; *p* ≤ 0.001 for the three parameters) (Fig. 7A–D). All these changes were significantly improved following treatment with either BUD, BUD-ES-NP + , or HA-BUD-ES-NP versus the positive control with *p* ≤ 0.001 regarding both DAI and ulcer score when comparing the three treated groups to the positive control one. However, when comparing the colon length in BUD, BUD-ES-NP + , and HA-BUD-ES-NP-treated groups versus the positive control one, *p* values were ≤ 0.05, ≤ 0.01, and ≤ 0.001, respectively. The highly significant changes observed indicated mucosal healing and alleviation of inflammation [52]. It is worth mentioning that HA-BUD-ES-NP presented the best recovery with *p* ˃ 0.05 regarding colon length and ulcer score when compared to the negative control group.

#### Histopathological changes assessment by H&E staining

**Fig. 8:**
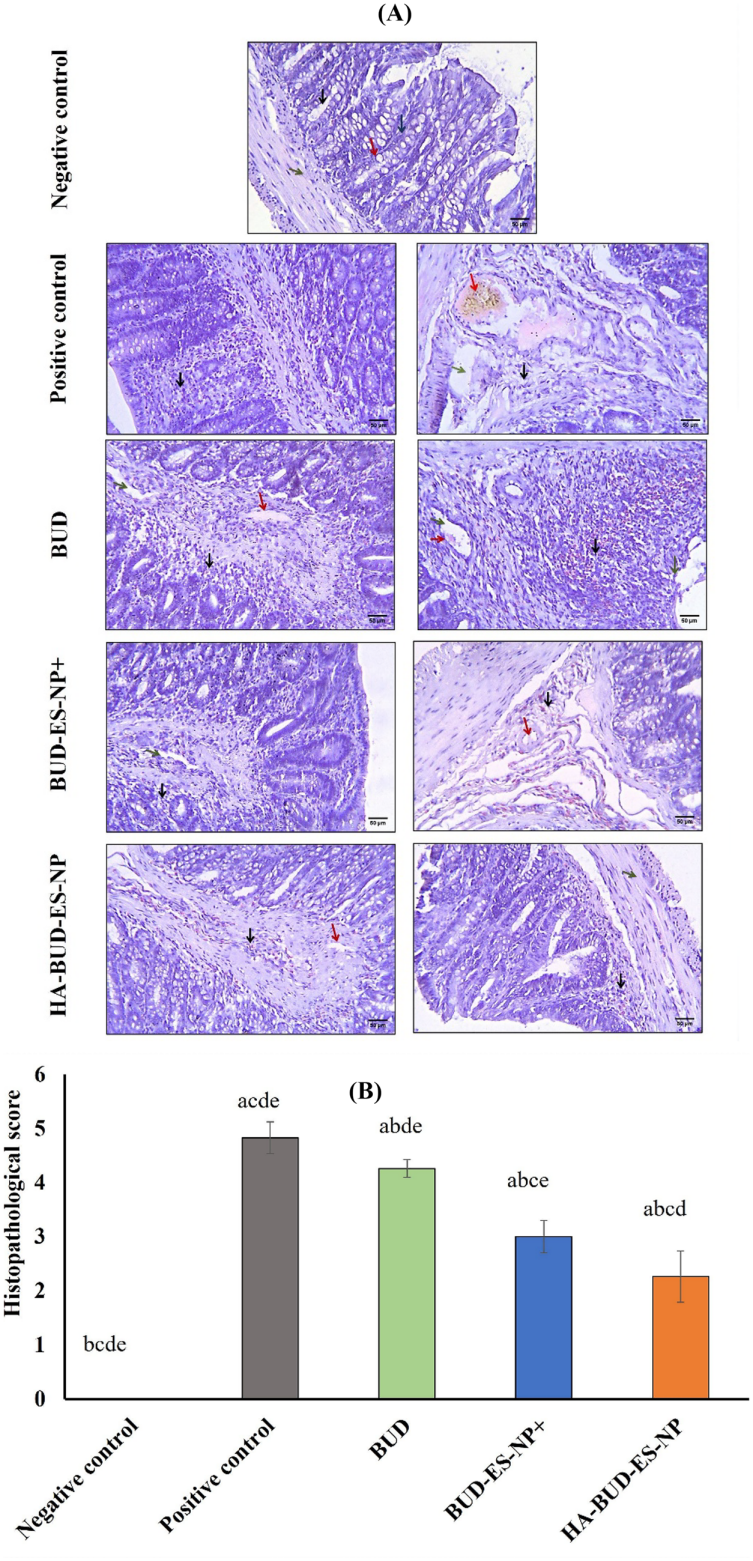
**A** Histopathological examination of H&E-stained colon tissue sections of negative control showing normal colonic mucosa. Regularly and parallelly arranged crypts (black arrows) perpendicular to the muscularis mucosae consisting of absorptive cells, goblet cells, and endocrine cells (red arrows). The lamina propria contains a variable number of inflammatory cells and a rich network of capillaries, venules, and lymphatics (blue arrows), and normal submucosa (green arrows). Positive control showed complete loss of normal structure of colonic mucosa with extensive necrotic destruction of the epithelium, hemorrhage, edema, crypt damage (red arrows), and ulceration and vacuolation at mucosal and sub-mucosal layers (green arrows), in addition to severe inflammatory cellular infiltration (black arrows). The BUD group showed loss of normal structure of colonic mucosa with necrotic destruction of the epithelium which is still seen. Also, some scattered hemorrhage, edema, crypt damage (red arrows), and small areas of ulceration and vacuolation at mucosal and sub-mucosal layers (green arrows), in addition to severe inflammatory cellular infiltration (black arrows). The BUD-ES-NP + group showed an almost normal structure of colonic mucosa with mild inflammatory cellular infiltration (black arrows) and a small area of ulceration and vacuolation (green arrows) and edema (red arrows) with almost normal crypts. HA-BUD-ES-NP showing normal colonic mucosa with a small area of ulceration and vacuolation (red arrows) and mild inflammatory cellular infiltration (black arrows) and normal submucosa (green arrows). The scale bar represents 50 µm. **B** The histopathological score of treated groups following administration of BUD, BUD-ES-NP + , and HA-BUD-ES-NP compared to control. Values were expressed as mean ± SD (*n* = 5). ^a^*p* ≤ 0.05 vs negative control, ^b^*p* ≤ 0.05 vs positive control, ^c^*p* ≤ 0.05 vs BUD, ^d^*p* ≤ 0.05 vs BUD-ES-NP + , and ^e^*p* ≤ 0.05 vs HA-BUD-ES-NP

Histopathological examination of H&E-stained colon tissue sections was done (Fig. 8A). In comparison to the normal histological appearance shown in the negative control group, the examination of positive control group sections revealed a complete loss of normal structure of colonic mucosa. Sections showed extensive necrotic destruction of the epithelium with hemorrhage, edema, crypt damage, ulceration, and vacuolation at mucosal and sub-mucosal layers. In addition, severe inflammatory cellular infiltration was also noted. The loss of normal colonic mucosal structure is still seen in sections from BUD-treated group. Areas of epithelial necrotic destruction, some scattered hemorrhage, edema, crypt damage, and small mucosal ulcers were still present. Moreover, cellular inflammatory infiltration was evident. On the other hand, BUD-ES-NP + and HA-BUD-ES-NP-treated groups showed substantial inflammation subsidence with the almost normal structure of colonic mucosa and mild inflammatory cellular infiltration. Small areas of ulceration, vacuolation, and edema were still seen. However, almost normal crypts and submucosa were seen in the HA-BUD-ES-NP-treated group.

Based on the above findings, a histopathological score was calculated (Fig. 8B). This was significantly increased in the positive control group (4.83 ± 0.29) versus the negative control one (*p* ≤ 0.001). Yet, it was significantly improved following treatment with either BUD (4.26 ± 0.16), BUD-ES-NP + (3 ± 0.29), or HA-BUD-ES-NP (2.26 ± 0.47) with *p* ≤ 0.001 for both BUD-ES-NP + and HA-BUD-ES-NP-treated groups versus the positive control.

#### Expression of inflammatory markers in colonic tissue homogenate

**Fig. 9:**
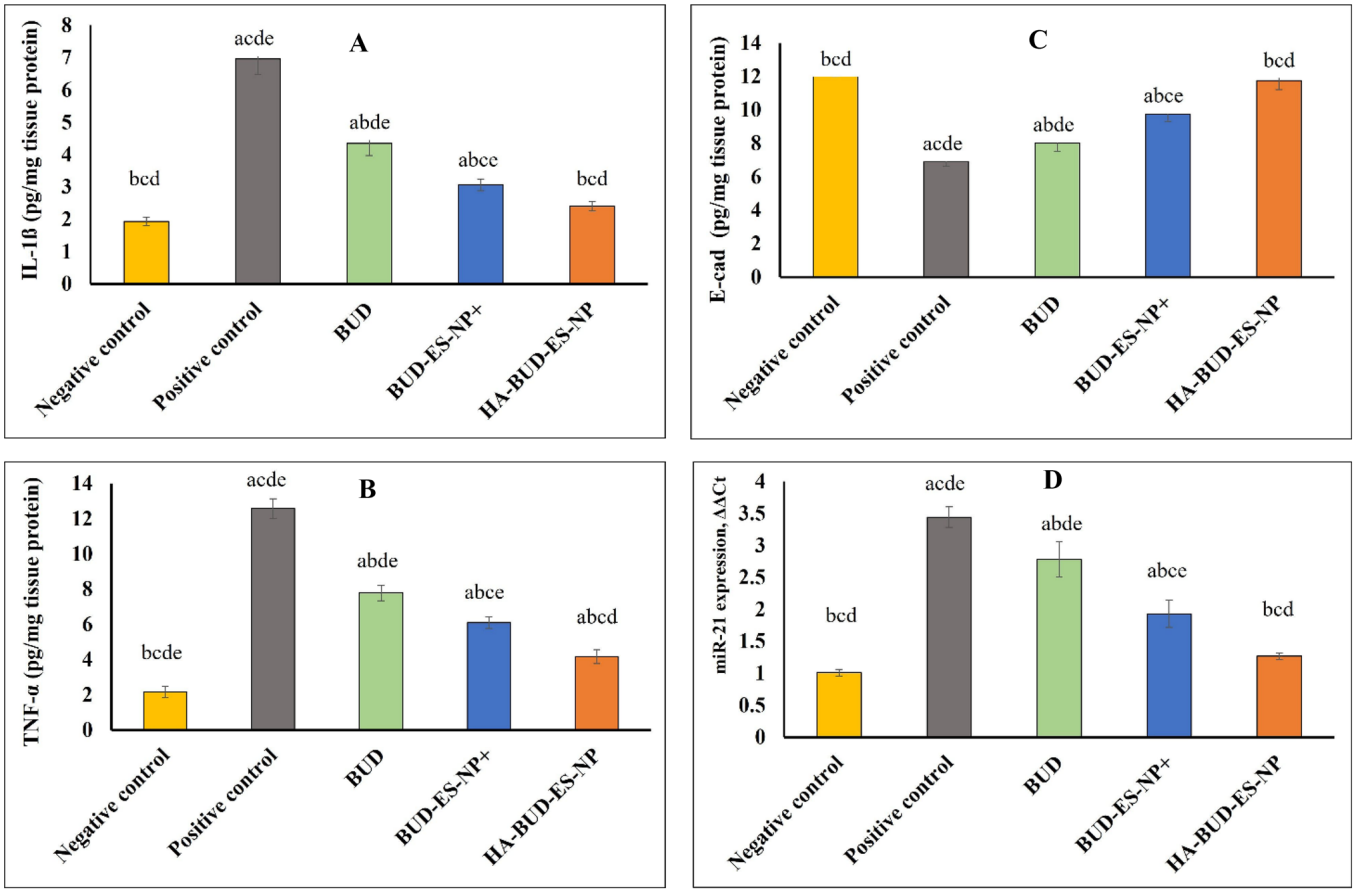
Evaluation of various biomarkers in colonic tissue; **A** interleukin-1ß (IL-1ß, **B** tumor necrosis factor-alpha (TNF-α), **C** colonic E-cadherin (E-cad), and **D** colonic miR-21 expression in acetic acid-induced colitis following administration of BUD, BUD-ES-NP + , and HA-BUD-ES-NP compared to control. Values were expressed as mean ± SD (*n* = 5). ^a^*p* ≤ 0.05 vs negative control, ^b^*p* ≤ 0.05 vs positive control, ^c^*p* ≤ 0.05 vs BUD, ^d^*p* ≤ 0.05 vs BUD-ES-NP + , and ^e^*p* ≤ 0.05 vs HA-BUD-ES-NP

Since inflammation plays a major role in the pathogenesis of acetic acid-induced colitis, the expression of inflammatory markers in colonic tissue homogenate was assessed. The selection of inflammatory biomarkers was based on previous studies, where TNF-α expression was found to accelerate the inflammatory cascade through nuclear factor (NF-κB) pathway activation and hence is directly involved in colon tissue destruction [53, 54]. Also, McAlindon et al. demonstrated the evident role of IL-1β in IBD [55]. The role of the selected inflammatory biomarkers was confirmed by the significant increase in IL-1β and TNF-α (*p* ≤ 0.05) in the positive control group versus the negative control one (2.6 and 4.8 folds increase, respectively) (Fig. 9A). This significant elevation further explains the marked inflammatory cellular infiltration observed in the histopathological sections of the positive control group (Fig. 8A). Treatment with BUD, BUD-ES-NP + , and HA-BUD-ES-NP led to 37%, 56%, and 65% decrease in IL-1β respectively when compared to the positive control group (Fig. 9A). A similar pattern was observed for TNF-α expression which also showed a significant decrease of 38%, 52%, and 69% following treatment with BUD, BUD-ES-NP + , and HA-BUD-ES-NP, respectively, compared to the positive control group (Fig. 9B).

#### Expression of colonic E-cadherin

There is a clear link between IBD etiology and deficits in gastrointestinal epithelial barrier function. An intact gut barrier protects against leakage of antigens from the intestinal lumen into the interstitial space and the lamina propria. It also controls polymorphonuclear leukocytes (PMN) migration across the epithelium into the lumen. A pivotal component of the epithelial adherens junction, E-cadherin, was shown to play a crucial role in cell–cell adhesions which are fundamental to intestinal epithelial barrier function [56]. Previous studies demonstrated mutations and changes in the E-cadherin gene in UC patients [57, 58]. Also, a dominant interfering E-cadherin mutant in the intestinal epithelium was shown in IBD-affected mice [59]. Moreover, the functional involvement of cytoplasmic E-cadherin-associated protein p120-catenin in IBD is well reported [60, 61]. In the current study, IBD induction by acetic acid resulted in a 45% decrease in E-cadherin in the positive control group versus the normal negative control one with *p* ≤ 0.001 (Fig. 9C). However, following treatment with BUD, BUD-ES-NP + , and HA-BUD-ES-NP, a respective increase of 16%, 41%, and 70% in E-cadherin level was observed compared to the positive control group (*p* ≤ 0.05 for BUD-treated group, *p* ≤ 0.001 for BUD-ES-NP + and HA-BUD-ES-NP-treated groups). Moreover, restoration of E-cadherin expression was achieved by HA-BUD-ES-NP showing an insignificant difference compared to its level in the negative control group (*p* > 0.05).

#### Colonic miR-21 expression

One of the most widely studied miRNAs regarding health and disease is miR-21. Regarding IBD, miR-21 level elevation is suggested to be a pathological finding [62, 63]. Our results showed that IBD induction by acetic acid led to a significant 2.4 folds increase in colonic miR-21 expression in the positive control group versus the normal negative control one with *p* ≤ 0.001 (Fig. 9D). This is in accordance with other studies showing that miR-21 ablation in mice is protective against DSS-induced colitis [64, 65]. Also, miR-21 downregulation was reported in UC patients in the remission phase [66]. However, treatment with BUD, BUD-ES-NP + , and HA-BUD-ES-NP led to 19%, 44%, and 63% significant decrease in colonic miRNA-21 expression in the three treated groups, respectively, when compared with the positive control group (*p* ≤ 0.001 for the three groups). Furthermore, miRNA-21 expression in HA-BUD-ES-NP-treated group was insignificantly different from its level in the negative control group with *p* > 0.05.

**Fig. 10:**
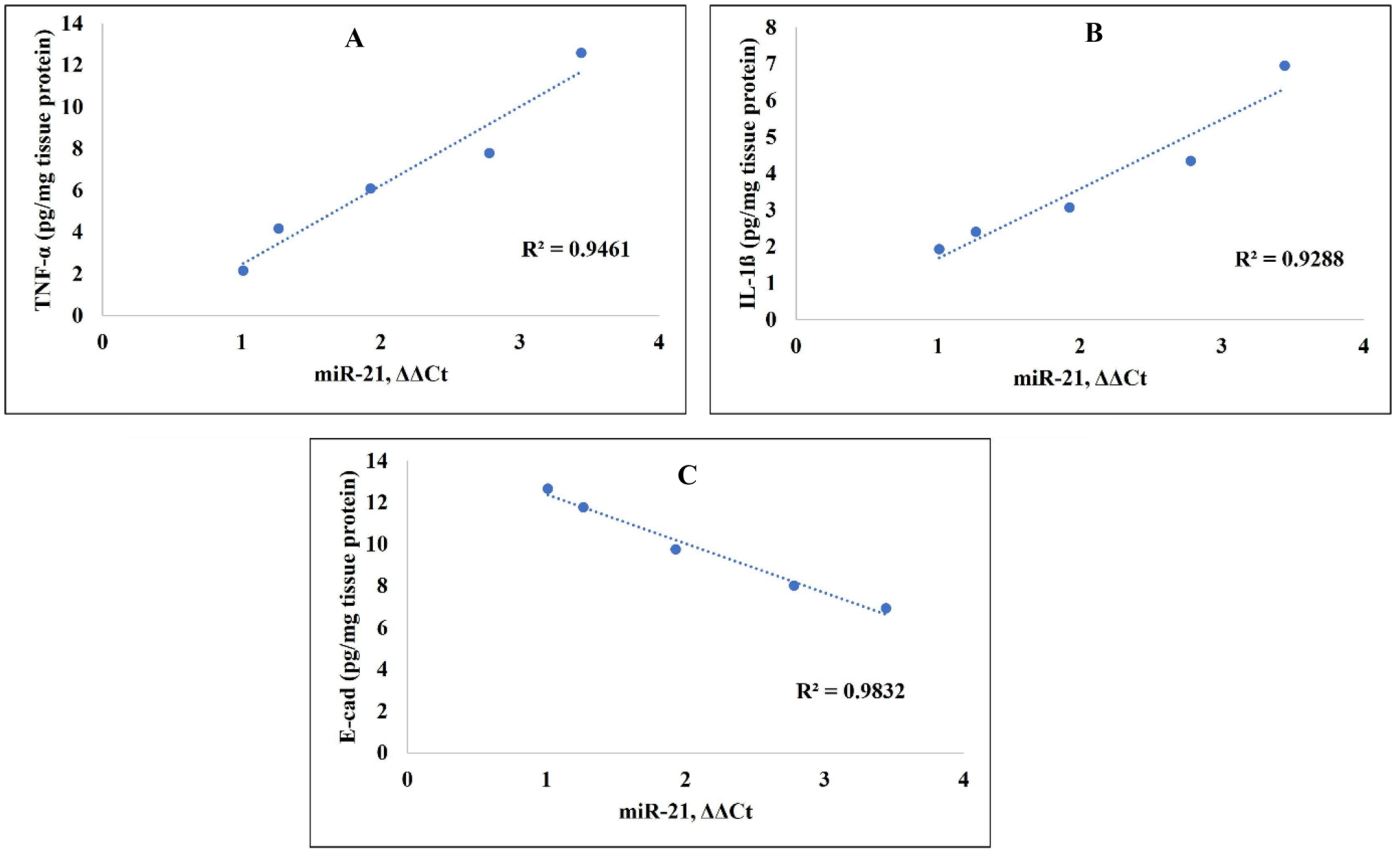
Correlation between levels of **A** and **B** inflammatory markers (TNF-α and IL-1ß) or **C** E-cadherin (E-cad) and miR-21 expression

The role of miR-21 in IBD pathogenesis could be attributed to its effect on intestinal inflammation. Herein, we found a strong positive correlation (*R*^2^ > 0.9) between miR-21 expression and inflammatory markers, TNF-α and IL-1β (Fig. 10A and B). In accordance with our results, the miR-21 expression on immune cells with the promotion of inflammatory cytokines production has been previously reported [67, 68]. Another possible effect of miR-21 in IBD pathogenesis is its effect on gut permeability where a negative correlation between E-cadherin and miR-21 levels was previously reported [69, 70]. Similarly, a strong negative correlation between colonic miR-21 expression and colonic E-cadherin level with a determination coefficient of 0.98 was observed in the current study (Fig. 10C). Recently, one study showed exacerbation of dextran sulfate sodium (DSS)-induced colitis in mice due to the uptake of exosome-derived miR-21a-5p from abnormally polarized macrophages by intestinal epithelial cells and a decline in E-cadherin level [71].

The combined results of the in vivo tests performed on the acetic acid-induced IBD animal model all showed the enhanced efficacy of BUD upon loading into NPs. Indeed, the small size of nanocarriers was previously shown to allow for more efficient drug targeting to affected tissues through the eEPR effect which allows for accretion at the inflamed and disrupted epithelium [72]. Also, inflamed tissues show epithelial barrier disruption, in addition, to an increase in the production of mucus and immune cell infiltration [73]. These were asserted as factors affecting the preferential uptake of NPs by inflamed cells and hence enhanced drug accumulation in damaged tissues [74]. In addition to the small size of NPs, two different approaches were exploited in the current study to improve the specificity of drug delivery towards colon inflammation sites. First, ES100, a methacrylic acid copolymer that takes advantage of pH changes along different regions of the GIT, was selected for the preparation of NPs. This probably allowed for pH-dependent release of BUD in the colon. Several studies demonstrated enhanced efficacy of NPs drug payload in various IBD animal models upon using ES100 either as a NP coat or in the matrix [14, 23, 75]. To further improve selectivity to inflammation, HA was used for NPs coating. HA-BUD-ES-NP showed superior efficacy compared to BUD and BUD-ES-NP + sometimes comparable to negative control as in colon length, ulcer score, histopathological findings, and restoration of level of different biomarkers tested. This could be explained by the ability of the negatively charged HA-coated NPs to preferentially adhere to inflamed colonic regions due to large amounts of positively charged proteins accumulated in inflamed mucosa [76]. Moreover, in inflamed IBD sites, endothelial cells express high levels of CD44 which is essential for inflamed tissues’ immune cell infiltration [77]. Hence, HA is considered an ideal moiety for targeted delivery to inflamed mucosa as it is well-reported to target colonic epithelial cells overexpressing CD44 and macrophages at inflamed colonic sites [47].

In conclusion, the developed HA-coated pH-sensitive NPs for the targeted BUD delivery to inflammation in IBD showed good colloidal properties with high entrapment efficiency and the ability to reduce early drug release in the stomach or small intestine. In vitro cell culture studies on Caco-2 cells and in vivo evaluation in an acetic acid-induced colitis model verified the augmented efficacy of HA-coated ES-NPs at both the cellular and molecular levels compared to BUD and the uncoated nanoparticles. Thus, our findings present HA-BUD-ES-NPs as a promising smart nanosystem with the capacity to promote mucosal healing and decrease inflammation for IBD treatment and a potential to be translated from bench to bedside.

## Data Availability

The authors confirm that the data for this study findings are available within the article and the supplementary information file.

## Declarations

### Animal studies

All institutional and national guidelines for the care and use of laboratory animals were followed.

## References

[1] Baumgart DC, Carding SR (2007). Inflammatory bowel disease: cause and immunobiology. Lancet.

[2] Abraham C, Cho JH (2009). Inflammatory bowel disease. N Engl J Med.

[3] Van Der Kraak L, Gros P, Beauchemin N (2015). Colitis-associated colon cancer: is it in your genes?. World J Gastroenterol.

[4] Ko JK, Auyeung KK (2014). Inflammatory bowel disease: etiology, pathogenesis and current therapy. Curr Pharm Des.

[5] Mentella MC, Scaldaferri F, Pizzoferrato M, Gasbarrini A, Miggiano GAD. Nutrition, IBD and gut microbiota: a review. Nutrients. 2020;12(4).10.3390/nu12040944. 10.3390/nu12040944 PMC7230231 32235316

[6] Stiegeler S, Mercurio K, Iancu MA, Corr SC. The impact of microRNAs during inflammatory bowel disease: effects on the mucus layer and intercellular junctions for gut permeability. Cells. 2021;10(12).10.3390/cells10123358. 10.3390/cells10123358 PMC8699384 34943865

[7] Cai Z, Wang S, Li J. Treatment of inflammatory bowel disease: a comprehensive review. Front Med (Lausanne). 2021;8:765474.10.3389/fmed.2021.765474. 10.3389/fmed.2021.765474 PMC8720971 34988090

[8] Gomollón F, Dignass A, Annese V, Tilg H, Van Assche G, Lindsay JO (2017). 3rd European evidence-based consensus on the diagnosis and management of Crohn’s disease 2016: part 1: diagnosis and medical management. J Crohns Colitis.

[9] Hamedani R, Feldman RD, Feagan BG. Review article: drug development in inflammatory bowel disease: budesonide--a model of targeted therapy. Aliment Pharmacol Ther. 1997;11 Suppl 3:98–107; discussion 107–8.10.1111/j.1365-2036.1997.tb00814.x. 10.1111/j.1365-2036.1997.tb00814.x 9467984

[10] Yasmin F, Najeeb H, Shaikh S, Hasanain M, Naeem U, Moeed A (2022). Novel drug delivery systems for inflammatory bowel disease. World J Gastroenterol.

[11] Hua S (2020). Advances in oral drug delivery for regional targeting in the gastrointestinal tract - influence of physiological, pathophysiological and pharmaceutical factors. Front Pharmacol.

[12] Lin M, Dong L, Chen Q, Xu H, Han X, Luo R, et al. Lentinan-based oral nanoparticle loaded budesonide with macrophage-targeting ability for treatment of ulcerative colitis. Front Bioeng Biotechnol. 2021;9: 702173. 10.3389/fbioe.2021.702173. 10.3389/fbioe.2021.702173 PMC8429481 34513811

[13] Sinhmar GK, Shah NN, Chokshi NV, Khatri HN, Patel MM (2018). Process, optimization, and characterization of budesonide-loaded nanostructured lipid carriers for the treatment of inflammatory bowel disease. Drug Dev Ind Pharm.

[14] Ali H, Weigmann B, Neurath MF, Collnot EM, Windbergs M, Lehr CM (2014). Budesonide loaded nanoparticles with pH-sensitive coating for improved mucosal targeting in mouse models of inflammatory bowel diseases. J Control Release.

[15] Collnot EM, Ali H, Lehr CM (2012). Nano- and microparticulate drug carriers for targeting of the inflamed intestinal mucosa. J Control Release.

[16] Schmidt C, Lautenschlaeger C, Collnot EM, Schumann M, Bojarski C, Schulzke JD (2013). Nano- and microscaled particles for drug targeting to inflamed intestinal mucosa: a first in vivo study in human patients. J Control Release.

[17] Vafaei SY, Abdolghaffari AH, Mahjub R, Eslami SM, Esmaeili M, Abdollahi M (2022). Budesonide-loaded hyaluronic acid nanoparticles for targeted delivery to the inflamed intestinal mucosa in a rodent model of colitis. Biomed Res Int.

[18] Barani M, Rahdar A, Sargazi S, Amiri MS, Sharma PK, Bhalla N. Nanotechnology for inflammatory bowel disease management: detection, imaging and treatment. Sens Bio-Sensing Res.2021;32:100417.10.1016/j.sbsr.2021.100417.

[19] Ansari F, Pourjafar H, Jodat V, Sahebi J, Ataei A (2017). Effect of Eudragit S100 nanoparticles and alginate chitosan encapsulation on the viability of Lactobacillus acidophilus and Lactobacillus rhamnosus. AMB Express.

[20] Anwer MK, Al-Shdefat R, Ezzeldin E, Alshahrani SM, Alshetaili AS, Iqbal M. Preparation, evaluation and bioavailability studies of Eudragit coated PLGA nanoparticles for sustained release of eluxadoline for the treatment of irritable bowel syndrome. 2021;8.10.3389/fphar.2017.00844. 10.3389/fphar.2017.00844 PMC5702012 29209215

[21] Subudhi MB, Jain A, Jain A, Hurkat P, Shilpi S, Gulbake A, et al. Eudragit S100 coated citrus pectin nanoparticles for colon targeting of 5-fluorouracil. 2015;8(3):832–849. 10.3390/ma8030832 PMC5455456 28787974

[22] Ibrahim B, Mady OY, Tambuwala MM, Haggag YA (2022). pH-sensitive nanoparticles containing 5-fluorouracil and leucovorin as an improved anti-cancer option for colon cancer. Nanomedicine (Lond).

[23] Qelliny MR, Aly UF, Elgarhy OH, Khaled KA (2019). Budesonide-loaded Eudragit S 100 nanocapsules for the treatment of acetic acid-induced colitis in animal model. AAPS PharmSciTech.

[24] Chiu CT, Kuo SN, Hung SW, Yang CY. Combined treatment with hyaluronic acid and mesalamine protects rats from inflammatory bowel disease induced by intracolonic administration of trinitrobenzenesulfonic acid. Molecules. 2017;22(6).10.3390/molecules22060904. 10.3390/molecules22060904 PMC6152619 28556814

[25] Filpa V, Bistoletti M, Caon I, Moro E, Grimaldi A, Moretto P (2017). Changes in hyaluronan deposition in the rat myenteric plexus after experimentally-induced colitis. Sci Rep.

[26] Yus C, Arruebo M, Irusta S, Sebastián V. Microflow nanoprecipitation of positively charged gastroresistant polymer nanoparticles of Eudragit^®^ RS100: a study of fluid dynamics and chemical parameters. 2020;13(13):2925. 10.3390/ma13132925 PMC7372341 32629799

[27] Radwan SE, El-Moslemany RM, Mehanna RA, Thabet EH, Abdelfattah EA, El-Kamel A (2022). Chitosan-coated bovine serum albumin nanoparticles for topical tetrandrine delivery in glaucoma: in vitro and in vivo assessment. Drug Deliv.

[28] Turanlı Y, Acartürk F. Preparation and characterization of colon-targeted pH/time-dependent nanoparticles using anionic and cationic polymethacrylate polymers. European J Pharm Sci. 2022;171:106122.10.1016/j.ejps.2022.106122. 10.1016/j.ejps.2022.106122 35007712

[29] Varshosaz J, Emami J, Tavakoli N, Minaiyan M, Rahmani N, Ahmadi F (2011). Development and validation of a rapid HPLC method for simultaneous analysis of budesonide and its novel synthesized hemiesters in colon specific formulations. Res Pharm Sci.

[30] Varshosaz J, Emami J, Tavakoli N, Minaiyan M, Rahmani N, Dorkoosh F (2012). Colon specific delivery of budesonide based on triple coated pellets: in vitro/in vivo evaluation. Acta Pharm.

[31] Wu X-X, Huang X-L, Chen R-R, Li T, Ye H-J, Xie W (2019). Paeoniflorin prevents intestinal barrier disruption and inhibits lipopolysaccharide (LPS)-induced inflammation in Caco-2 cell monolayers. Inflammation.

[32] El-Tanbouly GS, Abdelrahman RS (2022). The emerging coloprotective effect of sildenafil against ulcerative colitis in rats via exerting counterbalance between NF-κB signaling and Nrf-2/HO-1 pathway. Inflammopharmacology.

[33] Wallace JL, Keenan CM (1990). An orally active inhibitor of leukotriene synthesis accelerates healing in a rat model of colitis. Am J Physiol.

[34] Kumar N, Aggarwal R, Chauhan MK (2020). Extended levobunolol release from Eudragit nanoparticle-laden contact lenses for glaucoma therapy. Future J Pharm Sci.

[35] Rodrigues DF, Couto RO, Sinisterra RD, Jensen CE. Novel Eudragit^®^ -based polymeric nanoparticles for sustained release of simvastatin. Braz J Pharm Sci. 2020;56:e18363.10.1590/s2175-97902019000418363.

[36] Wang Y, Li M, Xu X, Tang W, Xiong L, Sun Q (2019). Formation of protein corona on nanoparticles with digestive enzymes in simulated gastrointestinal fluids. J Agric Food Chem.

[37] Zhang H, Wu T, Yu W, Ruan S, He Q, Gao H (2018). Ligand size and conformation affect the behavior of nanoparticles coated with in vitro and in vivo protein corona. ACS Appl Mater Interfaces.

[38] Win KY, Feng SS (2005). Effects of particle size and surface coating on cellular uptake of polymeric nanoparticles for oral delivery of anticancer drugs. Biomaterials.

[39] Zauner W, Farrow NA, Haines AM (2001). In vitro uptake of polystyrene microspheres: effect of particle size, cell line and cell density. J Control Release.

[40] Ravar F, Saadat E, Gholami M, Dehghankelishadi P, Mahdavi M, Azami S (2016). Hyaluronic acid-coated liposomes for targeted delivery of paclitaxel, in-vitro characterization and in-vivo evaluation. J Control Release.

[41] Shehata EM, Gowayed MA, El-Ganainy SO, Sheta E, Elnaggar YS, Abdallah OY. Pectin coated nanostructured lipid carriers for targeted piperine delivery to hepatocellular carcinoma. Int J Pharm. 2022;619:121712.10.1016/j.ijpharm.2022.121712. 10.1016/j.ijpharm.2022.121712 35367582

[42] Jiang L, Li X, Liu L, Zhang Q (2013). Thiolated chitosan-modified PLA-PCL-TPGS nanoparticles for oral chemotherapy of lung cancer. Nanoscale Res Lett.

[43] Kotla NG, Burke O, Pandit A, Rochev Y. An orally administrated hyaluronan functionalized polymeric hybrid nanoparticle system for colon-specific drug delivery. Nanomaterials (Basel). 2019;9(9).10.3390/nano9091246. 10.3390/nano9091246 PMC6780722 31480704

[44] Caradonna L, Amati L, Magrone T, Pellegrino NM, Jirillo E, Caccavo D (2000). Invited review: enteric bacteria, lipopolysaccharides and related cytokines in inflammatory bowel disease: biological and clinical significance. J Endotoxin Res.

[45] Tu J, Xu Y, Xu J, Ling Y, Cai Y (2016). Chitosan nanoparticles reduce LPS-induced inflammatory reaction via inhibition of NF-κB pathway in Caco-2 cells. Int J Biol Macromol.

[46] Fredin MF, Ulfhammer E, Rhedin M, Melgar S, Mellgard B, Peterson A (2005). Anti-inflammatory effects of budesonide in intestinal epithelial cells. Pharmacol Res.

[47] Kotla NG, Isa IL, Rasala S, Demir S, Singh R, Baby BV, et al. Modulation of gut barrier functions in ulcerative colitis by hyaluronic acid system. Adv Sci (Weinh). 2022;9(4):e2103189.DOI: 10.1002/advs.202103189. 10.1002/advs.202103189 PMC8811821 34761543

[48] Vafaei SY, Esmaeili M, Amini M, Atyabi F, Ostad SN, Dinarvand R (2016). Self assembled hyaluronic acid nanoparticles as a potential carrier for targeting the inflamed intestinal mucosa. Carbohyd Polym.

[49] Elshazly SM, Elhassanny AE, Mahmoud NM. Cilostazol protects against acetic acid-induced colitis in rats: possible role for cAMP/SIRT1 pathway. Eur J Pharmacol. 2020;881:173234.10.1016/j.ejphar.2020.173234. 10.1016/j.ejphar.2020.173234 32497625

[50] Zaghloul MS, Elshal M, Abdelmageed ME. Preventive empagliflozin activity on acute acetic acid-induced ulcerative colitis in rats via modulation of SIRT-1/PI3K/AKT pathway and improving colon barrier. Environ Toxicol Pharmacol. 2022;91:103833.10.1016/j.etap.2022.103833. 10.1016/j.etap.2022.103833 35218923

[51] Ahmed O, Farid A, Elamir A (2022). Dual role of melatonin as an anti-colitis and anti-extra intestinal alterations against acetic acid-induced colitis model in rats. Sci Rep.

[52] Naeem M, Lee J, Oshi MA, Cao J, Hlaing SP, Im E (2020). Colitis-targeted hybrid nanoparticles-in-microparticles system for the treatment of ulcerative colitis. Acta Biomater.

[53] Ansari MN, Rehman NU, Karim A, Soliman GA, Ganaie MA, Raish M, et al. Role of oxidative stress and inflammatory cytokines (TNF-α and IL-6) in acetic acid-induced ulcerative colitis in rats: ameliorated by Otostegia fruticosa. Life (Basel).2021;11(3).10.3390/life11030195. 10.3390/life11030195 PMC8001148 33802553

[54] Parameswaran N, Patial S (2010). Tumor necrosis factor-α signaling in macrophages. Crit Rev Eukaryot Gene Expr.

[55] McAlindon ME, Hawkey CJ, Mahida YR (1998). Expression of interleukin 1 beta and interleukin 1 beta converting enzyme by intestinal macrophages in health and inflammatory bowel disease. Gut.

[56] Bandyopadhyay C, Schecterson L, Gumbiner BM (2021). E-cadherin activating antibodies limit barrier dysfunction and inflammation in mouse inflammatory bowel disease. Tissue Barriers.

[57] U.I.G., Barrett, J.C., Lee, J.C., Lees, C.W., Prescott, N.J., Anderson, C.A., , Consortium (2009). Genome-wide association study of ulcerative colitis identifies three new susceptibility loci, including the HNF4A region. Nat Genet.

[58] Muise AM, Walters TD, Glowacka WK, Griffiths AM, Ngan BY, Lan H (2009). Polymorphisms in E-cadherin (CDH1) result in a mis-localised cytoplasmic protein that is associated with Crohn’s disease. Gut.

[59] Hermiston ML, Gordon JI (1995). Inflammatory bowel disease and adenomas in mice expressing a dominant negative N-cadherin. Science.

[60] Smalley-Freed WG, Efimov A, Burnett PE, Short SP, Davis MA, Gumucio DL (2010). p120-catenin is essential for maintenance of barrier function and intestinal homeostasis in mice. J Clin Invest.

[61] Smalley-Freed WG, Efimov A, Short SP, Jia P, Zhao Z, Washington MK, et al. Adenoma formation following limited ablation of p120-catenin in the mouse intestine. PLoS One. 20116(5):e19880.10.1371/journal.pone.0019880. 10.1371/journal.pone.0019880 PMC3096651 21611205

[62] Barrett JC, Hansoul S, Nicolae DL, Cho JH, Duerr RH, Rioux JD (2008). Genome-wide association defines more than 30 distinct susceptibility loci for Crohn’s disease. Nat Genet.

[63] Yang Y, Ma Y, Shi C, Chen H, Zhang H, Chen N (2013). Overexpression of miR-21 in patients with ulcerative colitis impairs intestinal epithelial barrier function through targeting the Rho GTPase RhoB. Biochem Biophys Res Commun.

[64] Shi C, Liang Y, Yang J, Xia Y, Chen H, Han H, et al. MicroRNA-21 knockout improve the survival rate in DSS induced fatal colitis through protecting against inflammation and tissue injury. PLoS One*.*2013;8(6):e66814.10.1371/journal.pone.0066814. 10.1371/journal.pone.0066814 PMC3691313 23826144

[65] Johnston DGW, Williams MA, Thaiss CA, Cabrera-Rubio R, Raverdeau M, McEntee C (2018). Loss of microRNA-21 influences the gut microbiota, causing reduced susceptibility in a murine model of colitis. J Crohns Colitis.

[66] Ando Y, Mazzurana L, Forkel M, Okazaki K, Aoi M, Schmidt PT (2016). Downregulation of microRNA-21 in colonic CD3+ T cells in UC remission. Inflamm Bowel Dis.

[67] Lu TX, Munitz A, Rothenberg ME (2009). MicroRNA-21 is up-regulated in allergic airway inflammation and regulates IL-12p35 expression. J Immunol.

[68] Sheedy FJ (2015). Turning 21: induction of miR-21 as a key switch in the inflammatory response. Front Immunol.

[69] Yan L, Cao R, Liu Y, Wang L, Pan B, Lv X (2016). MiR-21-5p links epithelial-mesenchymal transition phenotype with stem-like cell signatures via AKT signaling in keloid keratinocytes. Sci Rep.

[70] Guz M, Dworzanski T, Jeleniewicz W, Cybulski M, Kozicka J, Stepulak A (2020). Elevated miRNA inversely correlates with E-cadherin gene expression in tissue biopsies from Crohn disease patients in contrast to ulcerative colitis patients. Biomed Res Int.

[71] Lu J, Liu D, Tan Y, Deng F, Li R (2021). M1 macrophage exosomes MiR-21a-5p aggravates inflammatory bowel disease through decreasing E-cadherin and subsequent ILC2 activation. J Cell Mol Med.

[72] Lamprecht A (2010). Selective nanoparticle adhesion can enhance colitis therapy. Nat Rev Gastroenterol Hepatol.

[73] Lamprecht A, Yamamoto H, Takeuchi H, Kawashima Y (2005). Nanoparticles enhance therapeutic efficiency by selectively increased local drug dose in experimental colitis in rats. J Pharmacol Exp Ther.

[74] Nunes R, Neves JD, Sarmento B (2019). Nanoparticles for the regulation of intestinal inflammation: opportunities and challenges. Nanomedicine (Lond).

[75] Zeeshan M, Ali H, Khan S, Khan SA, Weigmann B (2019). Advances in orally-delivered pH-sensitive nanocarrier systems; an optimistic approach for the treatment of inflammatory bowel disease. Int J Pharm.

[76] Xiao B, Xu Z, Viennois E, Zhang Y, Zhang Z, Zhang M (2017). Orally targeted delivery of tripeptide KPV via hyaluronic acid-functionalized nanoparticles efficiently alleviates ulcerative colitis. Mol Ther.

[77] Marinho A, Nunes C, Reis S. Hyaluronic acid: a key ingredient in the therapy of inflammation. Biomolecules. 2021;11(10).10.3390/biom11101518. 10.3390/biom11101518 PMC8533685 34680150

